# Supplementary material for: The distribution of fitness effects among synonymous mutations in a gene under directional selection
Source: eLife. 2019 Jul 19;8:e45952. doi: 10.7554/eLife.45952 (PMC6692132; doi:10.7554/eLife.45952)
Supplement: Supplementary file 1. [file elife-45952-supp1.docx]

**Supplementary file 1.** Sequences of *gtsB* mutagenesis primers are found in Tables S1 and S2.

**Table S1. Site-directed mutagenesis reverse primers**. Reverse primers were paired with a conserved forward primer F2-gtsB-F (see Table 1) to amplify sequences from 715 base pairs upstream from *gtsB* to the *gtsB* codon targeted for mutation (indicated in the primer name).

BsaI restriction sites (underlined) mediate seamless ligation to products targeting the same codon generated with forward mutagenesis primers.

| **Name** | **Oligonucleotide sequence (5' to 3')** | **Name** | **Oligonucleotide sequence (5' to 3')** |
| --- | --- | --- | --- |
| R_M1 | ACTGCGGGTCTCTGGGAAAATCCAGTACAGGG | R_F112 | ACTGCGGGTCTCAGCCTTCACGACGGATTTTCTG |
| R_F7 | ACTGCGGGTCTCACACAGCAACAGAACTCATGGG | R_A122 | ACTGCGGGTCTCCCATCGGGTACAGGTAAATGGTG |
| R_S8 | ACTGCGGGTCTCTGAACACAGCAACAGAACTCATG | R_W132 | ACTGCGGGTCTCAGGCGGTACCGGTGACGATC |
| R_S11 | ACTGCGGGTCTCAGGCTTTGCTGAACACAGCAAC | R_L142 | ACTGCGGGTCTCGGCCCATGCCCGGGTTGAG |
| R_A10 | ACTGCGGGTCTCCTTTGCTGAACACAGCAACAG | R_E152 | ACTGCGGGTCTCCCCAGCCCCAGTCACGCAG |
| R_F13 | ACTGCGGGTCTCACGGCGAGGCTTTGCTGAAC | Rmod_P162 | ACTGCGGGTCTCTCGATCAGCCAGTCGAGACG |
| R_A15 | ACTGCGGGTCTCCATCGAACGGCGAGGCTTTG | R_I171 | ACTGCGGGTCTCTCACCAGGCAGTAGACCACG |
| R_Q17 | ACTGCGGGTCTCGCAGTGCATCGAACGGCG | R_M182 | ACTGCGGGTCTCTGATGAAGCCCGAGGCTTG |
| R_K22 | ACTGCGGGTCTCTGGGTAGCCAGCGCTGCAG | R_V192 | ACTGCGGGTCTCCGCCACGCAGGCCGGCGAG |
| R_P27 | ACTGCGGGTCTCGCGCCAGCACCAGTTTGG | R_I202 | ACTGCGGGTCTCTCTGTGCGGCACGGATGATC |
| R_V32 | ACTGCGGGTCTCCGATGAACATGCTGGGCG | R_W212 | ACTGCGGGTCTCAGTAGATGCGCGGCAGGC |
| R_Y37 | ACTGCGGGTCTCAAAAGCCCACCAACACGATG | R_V222 | ACTGCGGGTCTCCCGGACGCAGGCTTGGCAG |
| R_G38 | ACTGCGGGTCTCCGTAAAAGCCCACCAACACG | R_H232 | ACTGCGGGTCTCGCGCCAGGATCATCACCG |
| R_W42 | ACTGCGGGTCTCACAGGATGTAGCCGTAAAAGCCC | R_V241 | ACTGCGGGTCTCCCAGGTCGAAGCTCTTGATCG |
| R_S47 | ACTGCGGGTCTCACAGTACAAACGTCCACAGGATG | R_A242 | ACTGCGGGTCTCCCACCAGGTCGAAGCTCTTG |
| R_T52 | ACTGCGGGTCTCTCGATGTGGTGAACGACAGTAC | R_S252 | ACTGCGGGTCTCAGTAGCCCGGACCGCCGG |
| R_L62 | ACTGCGGGTCTCGCCCTGCCCATTTGTAACTCG | Rmod_S262 | ACTGCGGGTCTCGTACATGAACATCGCTGGCAGG |
| R_D72 | ACTGCGGGTCTCCGTTGTCGAACAAGCGCTC | R_M272 | ACTGCGGGTCTCTGCCCATCTGGCCACGGC |
| R_A82 | ACTGCGGGTCTCCCAGGTTTTTGCTCGCGAC | R_G282 | ACTGCGGGTCTCCGAGCATCAGGATTGCACTG |
| R_T92 | ACTGCGGGTCTCTGATACCGATAAACATCCCGCC | R_L292 | ACTGCGGGTCTCGGTAAGGCACGATGATCGCG |
| R_I101 | ACTGCGGGTCTCTCGCCAGTGTCACGCCGATG | R_D302 | ACTGCGGGTCTCCATTACGTTTGGTCCTCAGCTCG |
|  |  |  |  |

**Table S2. Site-directed mutagenesis forward primers**. Mutagenic forward primers were paired with a conserved reverse primer R3-gtsB-R (Table 1) to amplify sequences from the targeted codon (indicated in the primer name) to 173 base pairs downstream from *gtsB*. Mutations were introduced into oligonucleotides as 3-fold degenerate polymorphisms (bold and underlined). Primer names indicate codon and nucleotide positions targeted for mutation (ie, primer F_S8_2 targets the second position of the eighth codon, which encodes serine). BsaI restriction sites (underlined) mediate seamless ligation to products generated with reverse mutagenesis primers that target the same codon (or one codon prior for F2 primers) generated with reverse mutagenesis primers.

| **Name** | **Oligonucleotide sequence (5' to 3')** | **Name** | **Oligonucleotide sequence (5' to 3')** |
| --- | --- | --- | --- |
| F2_S2_1 | ACTGCGGGTCTCTTCCCATG**B**GTTCTGTTGCTGTGTTCAGCAAAG | F_L62_3 | ACTGCGGGTCTCCAGGGCT**V**GCACAGTACGAGCGCTTG |
| F2_S2_3 | ACTGCGGGTCTCTTCCCATGAG**V**TCTGTTGCTGTGTTCAGCAAAG | F_D72_2 | ACTGCGGGTCTCACAACG**B**TCGCTGGTGGGTCGCGAGC |
| F_F7_2 | ACTGCGGGTCTCCTGTGT**V**CAGCAAAGCCTCGCCGTTC | F_D72_3 | ACTGCGGGTCTCACAACGA**V**CGCTGGTGGGTCGCGAGC |
| F_F7_3 | ACTGCGGGTCTCCTGTGTT**D**AGCAAAGCCTCGCCGTTC | F_A82_1 | ACTGCGGGTCTCACCTG**H**CCTTGTTCGGCGGGATGTTT |
| F_S8_1 | ACTGCGGGTCTCTGTTC**B**GCAAAGCCTCGCCGTTCGATG | F_A82_3 | ACTGCGGGTCTCACCTGGC**D**TTGTTCGGCGGGATGTTT |
| F_S8_2 | ACTGCGGGTCTCTGTTCA**H**CAAAGCCTCGCCGTTCGATG | F_T92_2 | ACTGCGGGTCTCGTATCA**D**CTTGGTCATCGGCGTGACAC |
| F_S8_3 | ACTGCGGGTCTCTGTTCAG**D**AAAGCCTCGCCGTTCGATG | F_T92_3 | ACTGCGGGTCTCGTATCAC**D**TTGGTCATCGGCGTGACAC |
| F2_K9_1 | ACTGCGGGTCTCTGTTCAGC**B**AAGCCTCGCCGTTCGATGCAC | F2_F102_1 | ACTGCGGGTCTCTGGCGATC**V**TCCTCGACCAGAAAATCCGTCG |
| F2_K9_2 | ACTGCGGGTCTCTGTTCAGCA**B**AGCCTCGCCGTTCGATGCAC | F2_F102_3 | ACTGCGGGTCTCTGGCGATCTT**D**CTCGACCAGAAAATCCGTCG |
| F2_K9_3 | ACTGCGGGTCTCTGTTCAGCAA**B**GCCTCGCCGTTCGATGCAC | F_F112_2 | ACTGCGGGTCTCAAGGCT**V**TATCCGCACCATTTACCTGTAC |
| F_A10_1 | ACTGCGGGTCTCGCAAA**H**CCTCGCCGTTCGATGCACTG | F_F112_3 | ACTGCGGGTCTCAAGGCTT**V**ATCCGCACCATTTACCTGTAC |
| F_A10_2 | ACTGCGGGTCTCGCAAAG**D**CTCGCCGTTCGATGCACTG | F_A122_1 | ACTGCGGGTCTCCGATG**H**CGCTCTCGATGATCGTCACCG |
| F_A10_3 | ACTGCGGGTCTCGCAAAGC**D**TCGCCGTTCGATGCACTG | F_A122_3 | ACTGCGGGTCTCCGATGGC**H**CTCTCGATGATCGTCACCG |
| F_S11_1 | ACTGCGGGTCTCAAGCC**V**CGCCGTTCGATGCACTGCAG | F_W132_2 | ACTGCGGGTCTCCCGCCT**H**GAAATGGCTGCTCAACCCG |
| F_S11_2 | ACTGCGGGTCTCAAGCCT**D**GCCGTTCGATGCACTGCAG | F_W132_3 | ACTGCGGGTCTCCCGCCTG**H**AAATGGCTGCTCAACCCG |
| F_S11_3 | ACTGCGGGTCTCAAGCCTC**H**CCGTTCGATGCACTGCAG | F_L142_1 | ACTGCGGGTCTCTGGGC**D**TGGACAAACTCCTGCGTGACTG |
| F2_P12_1 | ACTGCGGGTCTCAAGCCTCG**D**CGTTCGATGCACTGCAGCGC | F_L142_3 | ACTGCGGGTCTCTGGGCCT**H**GACAAACTCCTGCGTGACTG |
| F2_P12_2 | ACTGCGGGTCTCAAGCCTCGC**D**GTTCGATGCACTGCAGCGC | F_E152_2 | ACTGCGGGTCTCGCTGGG**B**AGGCTTCCGTCTCGACTGG |
| F2_P12_3 | ACTGCGGGTCTCAAGCCTCGCC**H**TTCGATGCACTGCAGCGC | F_E152_3 | ACTGCGGGTCTCGCTGGGA**B**GGCTTCCGTCTCGACTGG |
| F_F13_1 | ACTGCGGGTCTCCGCCG**V**TCGATGCACTGCAGCGCTGG | Fmod_P162_1 | ACTGCGGGTCTCGATCGAC**D**CGGACCGCGTGGTCTACTGC |
| F_F13_2 | ACTGCGGGTCTCCGCCGT**V**CGATGCACTGCAGCGCTGG | Fmod_P162_3 | ACTGCGGGTCTCGATCGACCC**H**GACCGCGTGGTCTACTGC |
| F_F13_3 | ACTGCGGGTCTCCGCCGTT**D**GATGCACTGCAGCGCTGG | F2_A172_2 | ACTGCGGGTCTCTGGTGATCG**D**CGCCGTCTGGCAAGCCTCG |
| F2_D14_1 | ACTGCGGGTCTCCGCCGTTC**H**ATGCACTGCAGCGCTGGCTAC | F2_A172_3 | ACTGCGGGTCTCTGGTGATCGC**D**GCCGTCTGGCAAGCCTCG |
| F2_D14_2 | ACTGCGGGTCTCCGCCGTTCG**B**TGCACTGCAGCGCTGGCTAC | F_M182_1 | ACTGCGGGTCTCTCATC**B**TGGCGATGTTCCTCGCCGGC |
| F2_D14_3 | ACTGCGGGTCTCCGCCGTTCGA**V**GCACTGCAGCGCTGGCTAC | F_M182_3 | ACTGCGGGTCTCTCATCAT**H**GCGATGTTCCTCGCCGGC |
| F_A15_1 | ACTGCGGGTCTCTCGAT**H**CACTGCAGCGCTGGCTACC | F_V192_2 | ACTGCGGGTCTCGTGGCG**V**TGATCAATCGATCATCCGTGC |
| F_A15_2 | ACTGCGGGTCTCTCGATG**D**ACTGCAGCGCTGGCTACC | F_V192_3 | ACTGCGGGTCTCGTGGCGT**V**GATCAATCGATCATCCGTGC |
| F_A15_3 | ACTGCGGGTCTCTCGATGC**B**CTGCAGCGCTGGCTACC | F_I202_1 | ACTGCGGGTCTCCACAG**B**TCGACGGTGCGAGCCTGCCG |
| F2_L16_1 | ACTGCGGGTCTCTCGATGCA**D**TGCAGCGCTGGCTACCCAAAC | F_I202_3 | ACTGCGGGTCTCCACAGAT**D**GACGGTGCGAGCCTGCCG |
| F2_L16_2 | ACTGCGGGTCTCTCGATGCAC**V**GCAGCGCTGGCTACCCAAAC | F_W212_2 | ACTGCGGGTCTCTCTACT**H**GAGCGTGGTGCTGCCAAGC |
| F2_L16_3 | ACTGCGGGTCTCTCGATGCACT**H**CAGCGCTGGCTACCCAAAC | F_W212_3 | ACTGCGGGTCTCTCTACTG**H**AGCGTGGTGCTGCCAAGC |
| F_Q17_1 | ACTGCGGGTCTCCACTG**D**AGCGCTGGCTACCCAAACTG | F_V222_1 | ACTGCGGGTCTCGTCCG**H**TGTTCTTCAGTGCGGTGATGATC |
| F_Q17_2 | ACTGCGGGTCTCCACTGC**B**GCGCTGGCTACCCAAACTG | F_V222_3 | ACTGCGGGTCTCGTCCGGT**H**TTCTTCAGTGCGGTGATGATC |
| F_Q17_3 | ACTGCGGGTCTCCACTGCA**H**CGCTGGCTACCCAAACTG | F_H232_2 | ACTGCGGGTCTCTGGCGC**B**CATTGCGATCAAGAGCTTCG |
| F_K22_1 | ACTGCGGGTCTCTACCC**B**AACTGGTGCTGGCGCCCAGC | F_H232_3 | ACTGCGGGTCTCTGGCGCA**D**ATTGCGATCAAGAGCTTCG |
| F_K22_3 | ACTGCGGGTCTCTACCCAA**B**CTGGTGCTGGCGCCCAGC | F_V241_1 | ACTGCGGGTCTCACCTG**H**TGGCGGCGATGACGGCCGGC |
| F_P27_2 | ACTGCGGGTCTCTGGCGC**D**CAGCATGTTCATCGTGTTGGTG | F_V241_2 | ACTGCGGGTCTCACCTGG**V**GGCGGCGATGACGGCCGGC |
| F_P27_3 | ACTGCGGGTCTCTGGCGCC**D**AGCATGTTCATCGTGTTGGTG | F_V241_3 | ACTGCGGGTCTCACCTGGT**H**GCGGCGATGACGGCCGGC |
| F_V32_1 | ACTGCGGGTCTCTCATC**H**TGTTGGTGGGCTTTTACGGC | F_A242_1 | ACTGCGGGTCTCTGGTG**H**CGGCGATGACGGCCGGCGGTC |
| F_V32_3 | ACTGCGGGTCTCTCATCGT**H**TTGGTGGGCTTTTACGGC | F_A242_2 | ACTGCGGGTCTCTGGTGG**D**GGCGATGACGGCCGGCGGTC |
| F_Y37_1 | ACTGCGGGTCTCGCTTT**V**ACGGCTACATCCTGTGGACGTT | F_A242_3 | ACTGCGGGTCTCTGGTGGC**H**GCGATGACGGCCGGCGGTC |
| F_Y37_2 | ACTGCGGGTCTCGCTTTT**B**CGGCTACATCCTGTGGACGTT | F2_A243_1 | ACTGCGGGTCTCTGGTGGCG**H**CGATGACGGCCGGCGGTCCG |
| F_Y37_3 | ACTGCGGGTCTCGCTTTTA**D**GGCTACATCCTGTGGACGTT | F2_A243_2 | ACTGCGGGTCTCTGGTGGCGG**D**GATGACGGCCGGCGGTCCG |
| F_G38_1 | ACTGCGGGTCTCTTTAC**H**GCTACATCCTGTGGACGTTTGTAC | F2_A243_3 | ACTGCGGGTCTCTGGTGGCGGC**H**ATGACGGCCGGCGGTCCG |
| F_G38_2 | ACTGCGGGTCTCTTTACG**H**CTACATCCTGTGGACGTTTGTAC | F_S252_1 | ACTGCGGGTCTCGCTAC**V**CGTCCGACCTGCCAGCGATG |
| F_G38_3 | ACTGCGGGTCTCTTTACGG**D**TACATCCTGTGGACGTTTGTAC | F_S252_3 | ACTGCGGGTCTCGCTACTC**H**TCCGACCTGCCAGCGATG |
| F2_Y39_1 | ACTGCGGGTCTCTTTACGGC**V**ACATCCTGTGGACGTTTGTACTG | Fmod_S262_2 | ACTGCGGGTCTCATGTACT**D**GTTCACCTTCAGCCGTGGC |
| F2_Y39_2 | ACTGCGGGTCTCTTTACGGCT**B**CATCCTGTGGACGTTTGTACTG | Fmod_S262_3 | ACTGCGGGTCTCATGTACTC**H**TTCACCTTCAGCCGTGGC |
| F2_Y39_3 | ACTGCGGGTCTCTTTACGGCTA**D**ATCCTGTGGACGTTTGTACTG | F_M272_1 | ACTGCGGGTCTCTGGGC**B**TGGGCTCGGCCAGTGCAATC |
| F_W42_2 | ACTGCGGGTCTCTCCTGT**H**GACGTTTGTACTGTCGTTCACC | F_M272_3 | ACTGCGGGTCTCTGGGCAT**H**GGCTCGGCCAGTGCAATC |
| F_W42_3 | ACTGCGGGTCTCTCCTGTG**H**ACGTTTGTACTGTCGTTCACC | F_G282_2 | ACTGCGGGTCTCTGCTCG**H**TGCGATCCTCGCGATCATC |
| F_S47_1 | ACTGCGGGTCTCTACTG**V**CGTTCACCACATCGACGTTCC | F_G282_3 | ACTGCGGGTCTCTGCTCGG**V**GCGATCCTCGCGATCATC |
| F_S47_3 | ACTGCGGGTCTCTACTGTC**H**TTCACCACATCGACGTTCC | F_L292_1 | ACTGCGGGTCTCCTTAC**D**TCTATTCCGAGCTGAGGACCAA |
| F_T52_2 | ACTGCGGGTCTCCATCGA**D**GTTCCTGCCGAGTTACAAATGG | F_L292_3 | ACTGCGGGTCTCCTTACCT**D**TATTCCGAGCTGAGGACCAA |
| F_T52_3 | ACTGCGGGTCTCCATCGAC**H**TTCCTGCCGAGTTACAAATGG | F_D302_2 | ACTGCGGGTCTCGTAATG**B**CTAGTCTCGCCTCCAAACCTG |
| F_L62_1 | ACTGCGGGTCTCCAGGG**D**TTGCACAGTACGAGCGCTTG | F_D302_3 | ACTGCGGGTCTCGTAATGA**D**TAGTCTCGCCTCCAAACCTG |
|  |  |  |  |
