# Supplementary material for: The distribution of fitness effects among synonymous mutations in a gene under directional selection
Source: eLife. 2019 Jul 19;8:e45952. doi: 10.7554/eLife.45952 (PMC6692132; doi:10.7554/eLife.45952)
Supplement: Transparent reporting form [file elife-45952-transrepform.docx]

***eLife’s* transparent reporting form**

We encourage authors to provide detailed information *within their submission* to facilitate the interpretation and replication of experiments. Authors can upload supporting documentation to indicate the use of appropriate reporting guidelines for health-related research (see [EQUATOR Network](http://www.equator-network.org/%20)), life science research (see the [BioSharing Information Resource](https://biosharing.org/)), or the [ARRIVE guidelines](http://www.plosbiology.org/article/info:doi/10.1371/journal.pbio.1000412) for reporting work involving animal research. Where applicable, authors should refer to any relevant reporting standards documents in this form.

If you have any questions, please consult our Journal Policies and/or contact us: [editorial@elifesciences.org](mailto:editorial@elifesciences.org).

**Sample-size estimation**

- You should state whether an appropriate sample size was computed when the study was being designed
- You should state the statistical method of sample size computation and any required assumptions
- If no explicit power analysis was used, you should describe how you decided what sample (replicate) size (number) to use

Please outline where this information can be found within the submission (e.g., sections or figure legends), or explain why this information doesn’t apply to your submission:

In designing our study, we chose to introduce targeted mutations at every tenth codon along the gene and saturated the sites neighbouring three previously identified beneficial mutations. As discussed in the second paragraph of the Main Text section, we recovered 110 point mutants at 34 sites spanning the gene. This information is also shown in Figure 1. We did not include an explicit power analysis, as our sample size is similar to or greater than that tested in previous similar studies of point mutants.

**Replicates**

- You should report how often each experiment was performed
- You should include a definition of biological versus technical replication
- The data obtained should be provided and sufficient information should be provided to indicate the number of independent biological and/or technical replicates
- If you encountered any outliers, you should describe how these were handled
- Criteria for exclusion/inclusion of data should be clearly stated
- High-throughput sequence data should be uploaded before submission, with a private link for reviewers provided (these are available from both GEO and ArrayExpress)

Please outline where this information can be found within the submission (e.g., sections or figure legends), or explain why this information doesn’t apply to your submission:

This information can be found in the Materials and Methods section, specifically under the “Competitions” subheading, as well as in Figure 2. Exclusion of nonsense mutations is explained in Materials and Methods, subheading “Comparison and characterization of DFEs”.

As we encountered no outliers, this point does not apply to our submission.

**Statistical reporting**

- Statistical analysis methods should be described and justified
- Raw data should be presented in figures whenever informative to do so (typically when N per group is less than 10)
- For each experiment, you should identify the statistical tests used, exact values of N, definitions of center, methods of multiple test correction, and dispersion and precision measures (e.g., mean, median, SD, SEM, confidence intervals; and, for the major substantive results, a measure of effect size (e.g., Pearson's r, Cohen's d)
- Report exact p-values wherever possible alongside the summary statistics and 95% confidence intervals. These should be reported for all key questions and not only when the p-value is less than 0.05.

Please outline where this information can be found within the submission (e.g., sections or figure legends), or explain why this information doesn’t apply to your submission:

Reporting of statistical tests is included in the Main Text (page 2, lines 4-15; p. 3, ll. 6-10). Detailed methods for statistical analysis can be found in the Materials and Methods section, under the subheadings “Comparison and characterization of DFEs”, “Phylogeny construction”, and “Statistical analysis”.

Exact p-values are included throughout the main text.

(For large datasets, or papers with a very large number of statistical tests, you may upload a single table file with tests, Ns, etc., with reference to sections in the manuscript.)

**Group allocation**

- Indicate how samples were allocated into experimental groups (in the case of clinical studies, please specify allocation to treatment method); if randomization was used, please also state if restricted randomization was applied
- Indicate if masking was used during group allocation, data collection and/or data analysis

Please outline where this information can be found within the submission (e.g., sections or figure legends), or explain why this information doesn’t apply to your submission:

This information does not pertain to our submission, as culture conditions were kept constant across all mutants for each type of assay. There were no experimental groups.

**Additional data files (“source data”)**

- We encourage you to upload relevant additional data files, such as numerical data that are represented as a graph in a figure, or as a summary table
- Where provided, these should be in the most useful format, and they can be uploaded as “Source data” files linked to a main figure or table
- Include model definition files including the full list of parameters used
- Include code used for data analysis (e.g., R, MatLab)
- Avoid stating that data files are “available upon request”

Please indicate the figures or tables for which source data files have been provided:

Source data is available for figures 1, 2, and 3, whereas the experimental fitness data used in Figure 4 is included in the source data file for Figure 1. Code used for characterization of DFEs (Figure 1), testing fitness vs YFP expression (Figure 2C), testing promoter strength (Figure 3A), and estimating signal across the phylogeny (Figure 4) are also included.
